# An Ultrasensitive Electrochemical Immunosensor for HIV p24 Based on Fe_3_O_4_@SiO_2_ Nanomagnetic Probes and Nanogold Colloid-Labeled Enzyme–Antibody Copolymer as Signal Tag

**DOI:** 10.3390/ma6041255

**Published:** 2013-03-25

**Authors:** Ning Gan, Xiaowen Du, Yuting Cao, Futao Hu, Tianhua Li, Qianli Jiang

**Affiliations:** 1 The State Key Laboratory Base of Novel Functional Materials and Preparation Science, Faculty of Material Science and Chemical Engineering, Ningbo University, Ningbo 315211, China; E-Mails: duxiaowen@126.com (X.D.); hufutao@nbu.edu.cn (F.H.); litianhua@nbu.edu.cn (T.L.); 2 Department of Laboratory Medicine, Nanfang Hospital, Southern Medical University, Guangzhou 510515, China; E-Mail: jiangqianlid@yahoo.cn

**Keywords:** Fe_3_O_4_@SiO_2_ magnetic beads, nanogold-labeled EnVision antibody enzyme complex, HIV p24, signal amplification, electrochemical immunosensor

## Abstract

An ultrasensitive portable electrochemical immunosensor for human immunodeficiency virus p24 (HIV p24) antigen detection has been developed, whereby the detection sensitivity was 1000 times higher than that of the ELISA method. Firstly, a novel HRP enzyme–antibody copolymer (EV-p24 Ab2) was synthesized through an EnVision regent (EV, a dextrin amine skeleton anchoring more than 100 molecules of HRP and 15 molecules of anti IgG), then incubated in the secondary antibody of p24. Secondly, the copolymer was immobilized on the gold nanocolloids (AuNPs) to fabricate a novel signal tag (AuNPs/EV-p24 Ab2). Subsequently, a sandwich-type immunoreaction would take place between the capture probe (silicon dioxide-coated magnetic Fe_3_O_4_ nanoparticles (MNPs) labeled with the primary p24 antibody (MNPs-p24 Ab1)), p24 (different concentrations) and the signal tag [AuNPs/EV-p24 Ab2)] to form the immunocomplex. Finally, the immunocomplex was absorbed on the surface of screen printed carbon electrode (SPCE) by a magnet and immersed in the o-hydroxyl phenol (HQ) and H_2_O_2_. The large amounts of HRP on the signal tag can catalyze the oxidation of HQ by H_2_O_2_, which can induce an amplified reductive current. Moreover, the capture probe could improve the accumulation ability of p24 and facilitate its separation from the substrate through the magnet. Under optimal conditions, the proposed immunoassay exhibited good sensitivity to p24 within a certain concentration range from 0.001 to 10.00 ng/mL, with a detection limit of 0.5 pg/mL (S/N = 3). The proposed method can be used for real-time and early detection of HIV-infected people.

## 1. Introduction

The HIV-1 (human immunodeficiency virus type 1) capsid protein, the p24 antigen (p24), has great significance for diagnostics, because it can be detected several days earlier than host-generated HIV antibodies (which are the target of almost all current tests used in the field), and can be used to design sensitive assays without the need for polymerase chain reaction (PCR) [1]. Thus, it was very important to establish a highly sensitive detection method for p24 to predict AIDs diseases [2,3,4]. To date, various methods and strategies have been reported for determination of p24, such as radioimmunoassay [5], fluoroimmunoassay [6], enzyme-linked immunosorbent assay [7], and electrochemical methods [8,9], *etc.* However, these methods have many drawbacks, such as complex operating procedures, long analysis times, and expensive instruments. For example, the conventional sandwich-type ELISA is one of the major analytical techniques used for detection of p24 [8]. However, it is limited by its sensitivity and selectivity. Therefore, to develop simple, rapid, highly sensitive and selective detection techniques for the sensitive profiling of HIV, a method using the p24 antigen is still in critical demand. In order to achieve this goal, the fabrication of an ultrasensitive p24 immunosensor based on specific antigen–antibody interactions has become a priority.

Presently, based on the detection principle, the immunosensors can be categorized as follows: electrochemical, optical, or microgravimetric [10]. Compared to the optical immunosensor, the electrochemical immunosensor (ECI) is attracting more attention from researchers due to its low cost, wide dynamic concentration response range, high sensitivity, simple instrumentation, stability and versatility. Several electrochemical immunosensors have been developed to determine HIV p24 [1,2,8,9]. However, when ECI is applied to clinical diagnosis, the major shortcoming was that the sensor is still labor intensive, and needs 2–3 h for pre-enrichments of the ultra trace level of p24 from serum samples before analysis. Thus, it was very important to develop a robust pretreatment method against the interference effects of the complex biological matrix in serum.

The immune magnetic beads (IMBs) were widely used in enrichment and separation of particular protein in biology samples [11,12]. Recently, hybrid nano-IMBs, consisting of two or more different nano-scale functionalities, have attracted much attention due to their novel combined properties and multiple potential applications [13,14,15], among which Fe_3_O_4_(core)/SiO_2_(shell) nanoparticles (MNPs) have captured particular attention for immobilizing antibodies and enzymes as a result of its easy preparation of labeled bioconjugates and magnetic separation of antibody from unbound proteins [16,17]. Additionally, the SiO_2_ shell can also provide several anchoring sites for bimolecular immobilization through Si–OH. Therefore, MNPs have been applied in increasingly more areas such as immunoassays, protein immobilization, cell purification and magnetically controlled transport of anticancer drugs.

Signal amplification has been used extensively for the development of ultrasensitive amperometric immunoassay methods. In order to meet the increasing demand for early and ultrasensitive detection of tumor markers, three primary signal amplification strategies have been developed [13]. The first method involves the use of metal and semiconductor nanoparticles directly as electro-active labels to amplify the electrochemical detection of proteins [18,19]. The second method utilizes nanoparticles as carriers for the loading of a large amount of electro active species to amplify the detection signal [20,21,22]. The third method was the most extensively employed, and it uses enzyme-functionalized nanoparticles as labels. Enhanced sensitivity has been achieved by loading a large amount of enzyme for an individual sandwich immunological reaction event. Because of the outstanding optical and electronic performance and biocompatibility, the usage of a polymer to immobilize an enzyme, such as horseradish peroxidase (HRP), as a signal amplifier had been of particular interest in biosensor design.

The EnVision reagent (EV) is a kind of enzyme–polymer complex which contains about 100 molecules of HRP and 15 molecules of anti-IgG antibody connected in a poly-dextrin amine skeleton [23,24]. When EV is incubated with the secondary antibody of p24, the resulted copolymers (EV-p24 Ab2) can act as a very sensitive signal tag for the immunosensor because of its high enzyme-labeled density. With the development of nanotechnology, various types of nanomaterials have been applied in the fabrication of immunosensors, such as metal nanoparticles. Nanogold colloids (AuNPs) are a good matrix for the immobilization of enzyme and antibody [14]. The probes based on detection antibodies labeled on AuNPs have widely been used in clinical diagnosis. If AuNPs are employed as a matrix to immobilize more than one EV-p24 Ab2 on it as signal tag, the prepared AuNPs/EV-p24 Ab2 bioconjugates will have much higher labeled density of HRP enzymes than EV-p24 Ab2, which can further amplify the detection current. However, there is currently no report on the actual application of AuNPs-labeled EV copolymer as signal tag in the construction of electrochemical immunosensor.

In this paper, we have successfully designed an ultrasensitive ECI immunosensor for p24 detection using AuNPs/EV-p24 Ab2 bioconjugates as a signal tag and MNPs-p24 Ab1 as the capture probes. AuNPs/EV-p24 Ab2 could effectively amplify the catalytic current and the capture probes can simplify the pre-enrichment steps for extracting the ultra trace level of p24 in serums from a complex matrix. Based on ultrasensitive p24 detection, which was achieved to a detection limit of 1 pg/mL, the immunosensor exhibited simple instrumentation, high sensitivity, a wide linear range, good stability and specificity, and provides an excellent prospect for HIV clinical analysis.

## 2. Results and Discussion

### 2.1. Morphological Characterization of the Capture Probes and Signal Tag

**Figure 1:**
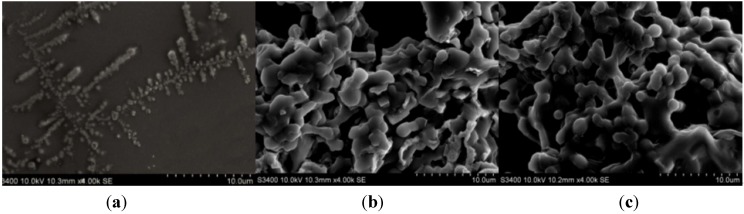
Scanning electron microscopy (SEM) images of (**a**) EnVision reagent (EV); (**b**) EV-p24 Ab2 and (**c**) Au NPs/EV-p24 Ab2.

Different AuNPs/EV-p24 Ab2 bioconjugates were characterized by scanning electron microscopy (SEM), as depicted in Figure 1. In Figure 1a, we can see EV has a tree-like skeleton shape with a large amount of HRP (island shapes) immobilized on it. After p24 Ab2 was conjugated to the EV, the EV-p24 Ab2 copolymer was visualized like a covering of scales, indicating that p24 Ab2 was connected to the skeleton of EV (Figure 1b). After AuNPs was employed as a matrix to immobilize EV-p24 Ab2 to produce the signal tag, many rounded masses or protuberances appeared (Figure 1c), which suggested that many AuNPs were embedded in the protein layers.

**Figure 2:**
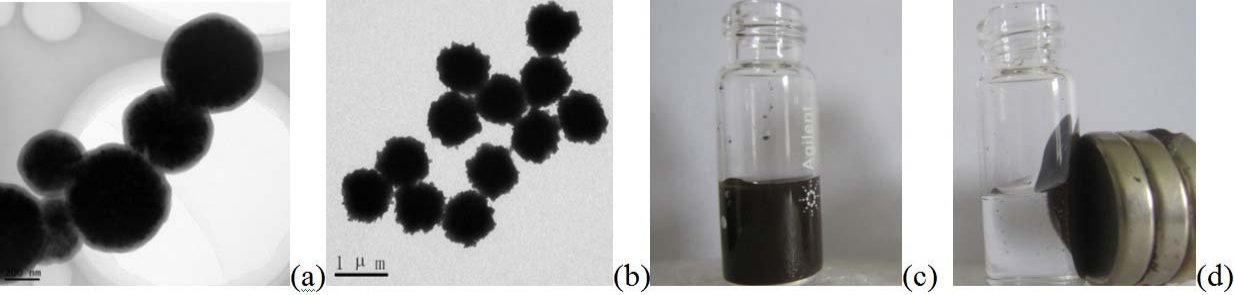
Transmission electron microscopy (TEM) images of (**a**) Fe_3_O_4_@SiO_2_ (MNPs) and (**b**) MNPs-p24 Ab1 and (**c**) MNPs-p24 Ab1 capture probes in the absence and (**d**) presence of an external magnetic field (**d**, right).

The transmission electron microscopy (TEM) image of the Fe_3_O_4_@SiO_2_ (MNPs) (Figure 2a) revealed that the MNPs have a diameter of about 200 ± 23 nm, and the SiO_2_ shell was about 10 ± 1 nm. Figure 2b showed that when the MNPs were labeled with p24 Ab1 to prepare the capture probe—MNPs-p24 Ab1—the size of the spherical particle enlarged to about 1 μm, indicating that the p24 Ab1 protein had been modified by the SiO_2_ shell. It can be concluded that an immune-complex formed. Figure 2c,d, respectively, reflect how the capture probes were in the absence and presence of a magnetic field, which showed that the capture probe has super paramagnetism.

**Figure 3:**
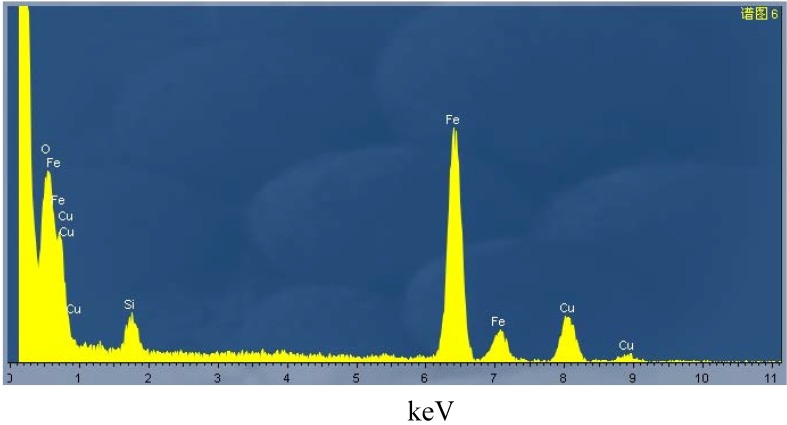
The X-ray fluorescent spectroscopic (XRF) spectrum of MNPs.

Figure 3 showed an X-ray fluorescent spectroscopic (XRF) spectrum of the MNPs particles set on a copper plate base. The characteristic peak for Si appeared at 1.75 eV and O at 0.65 eV. The characteristic peak for Fe at 6.43 and 7.15 eV can also be found. These proved that Fe_3_O_4_/SiO_2_ was successfully synthesized. X-ray fluorescent spectroscopic (XRF) analysis was also employed to demonstrate AuNPs/EV- p24 Ab2 signal tag. The characteristic peaks for gold element (Au 4s, Au 4p, Au 4d and Au 4f) appeared between 80 and 90 eV. The intensity of the characteristic peak for sulfur, S 1s, at 2.4 eV increased after thiolated p24 Ab2; more sulfur element was thus immobilized by the AuNPs.

**Figure 4:**
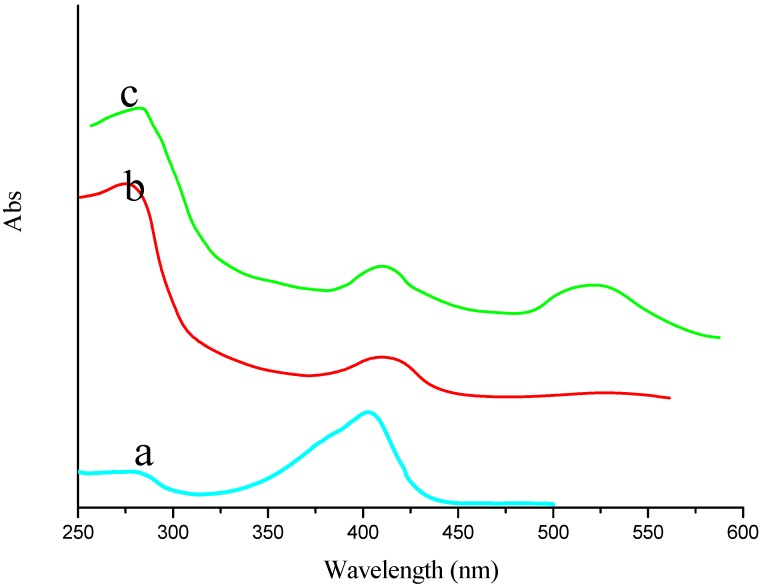
The ultraviolet-visible absorption spectrometry of (**a**) EV; (**b**) EV-p24 Ab2 and (**c**) Au/EV-p24 Ab2 signal tag, respectively.

Figure 4 showed the ultraviolet-visible absorption spectrometry (UV-VIS) for characterization of EV, EV-p24 Ab2 and AuNPs/EV-p24 Ab2. EV showed one peak at 410 nm at Figure 4a, which was attributed to HRP. Figure 4b showed EV-p24 Ab2 had two absorption bands, one was at 280 nm and attributed to p24 Ab2 (protein’s characteristic absorption peak), the other was at 410 nm and attributed to HRP (see trace “a” obtained for EV alone). Figure 4c showed another 520 nm peak which was attributed to AuNPs. From the results above, it can be concluded that AuNPs/EV-p24 Ab2 signal tag had been constructed successfully (Figure 4c).

### 2.2. Electrochemical Characterization of the Immunosensor

**Figure 5:**
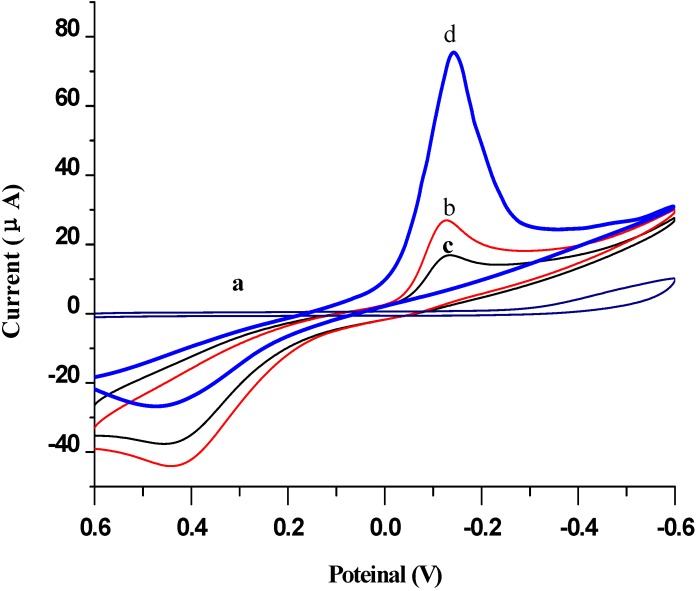
Cyclic voltammograms obtained at (**a**) SPCE/MNPs-p24 Ab1 electrode in pH 7.0 PBS; (**b**) SPCE/MNPs-p24 Ab1; (**c**) SPCE/MNPs-p24 Ab1 incubated in 5 ng/mL p24; and (**d**) c was further sandwich immunoreacted in Au NPs/EV-p24 Ab_2_ in pH 7.0 PBS containing 5 mmol/L HQ and H_2_O_2_.

The electrochemical characterization of the immunosensor was shown in Figure 5. The cyclic voltammograms of the MNPs-p24 Ab1 capture probe modified SPCE electrode by magnet (SPCE/MNPs-p24 Ab1) and did not show any signal in pH 7.0 PBS (curve a). Upon adding 10 μL PBS buffer solution with 5 mmol/L HQ and 5 mmol/L H_2_O_2_, the electrode exhibited a pair of stable and redox peaks, respectively, at −145 mV and 420 mV (curve b) of the redox probe of HQ. When the immunosensor was incubated with 5 ng/mL p24 antigen, both oxidation and reduction peaks were reduced (curve c), it was because the MNPs-p24 Ab1/p 24 immunocomplex did not have electrical activity and can hinder the electronic delivery of HQ. However, after it was further incubated with the AuNPs/EV-p24 Ab2 signal tag, an increase in the reduction current and a decrease in the oxidation current for the electrode could be observed, indicating that the reduction of H_2_O_2_ was catalyzed by HRP labeled on the signal tag, which has many HRP in the EV chain [25]. Due to its highly specific surface area, it may enhance the immobilized capacity toward the EV possessing a large amount of HRP, which favors the oxidation of HQ by H_2_O_2_ and hence provides a greatly amplified signal. Moreover, the signal amplification was also due to the excellent electrical conductivity of the AuNPs.

### 2.3. Optimization of Experimental Conditions

The volume and concentration of the capture probes (MNPs/p24 Ab1) highly influenced the fabrication performance of the immunosensor. To maintain its optimum film-forming ability on the surface of SPCE electrode, 10 µL of MNPs/p24 Ab1 composite solution was selected as the optimal amount dropped on the electrode surface. Furthermore, based on the ECL experimental results, 1.0 mg/mL capture probes was chosen as the optimal concentration.

**Figure 6:**
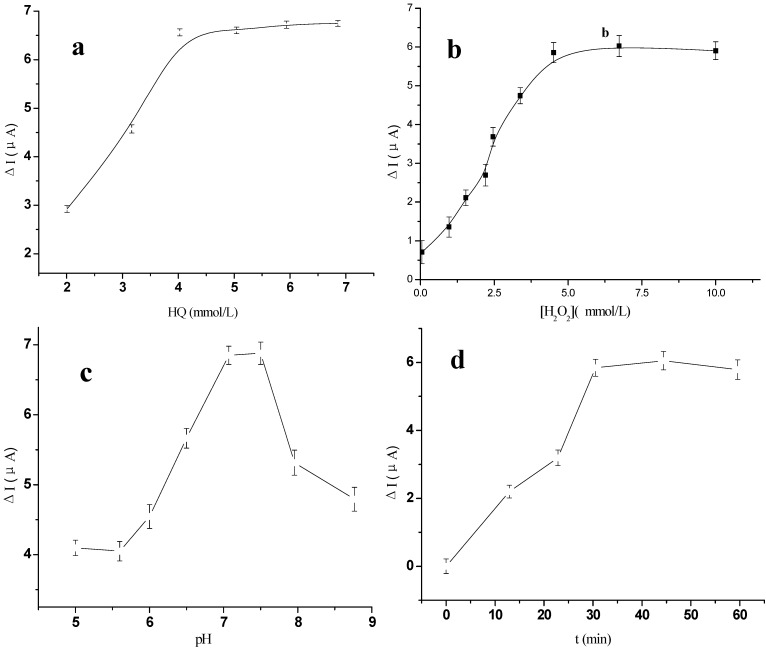
The effect of different (**a**) concentration of HQ; (**b**) Concentration of H_2_O_2_; (**c**) pH to the anodic peak currents responses for the immunosensor; (**d**) Incubation time after the immunosensor was incubated with 0.5 ng/mL p24 solution.

The immunoassay conditions, such as the concentration of HQ and H_2_O_2_, pH of the supporting electrolyte, incubation temperature and the incubation time, can also affect the sensor’s catalytic current. As illustrated in Figure 6a,b, the concentration of HQ and H_2_O_2_ was respectively optimized by varying its concentration. The current value increased to a maximum when both the concentration of HQ and H_2_O_2_ reached 5 mmol/L. The pH of the background solution could greatly affect the catalytic current signal of the immunosensor, because the activity of the immobilized protein may be influenced by the acidity of the solution, thus the effect of pH from 6.0 to 8.0 on the immunosensor performance was investigated using 5 ng/mL p24 solution. As shown in Figure 6c, the maximum catalytic current ∆*I* could be obtained at pH 7.0, where ∆*I* = *I*_s_ − *I*_0_, and *I*_0_ was the background reduction intensity of the immunosensor in HQ; and *I*_s_ was the catalytic current signal of the sandwich immunocomplex formed. Thus, the detection of p24 was performed in pH 7.0 PBS containing 5 mmol/L HQ and 5 mmol/L H_2_O_2_ throughout the experiment.

The effects of incubation temperature and incubation time on the ECL intensity of the immunosensor were also investigated. The ∆*I* increased with the increase of incubation temperature from 20 to 45 °C and a maximum ∆*I* was obtained at 37 °C. Figure 6d showed that ∆*I* increased with the increase of incubation time and reached a plateau at 30 min (the cumulative time for the electrode incubated in p24 and then in AuNPs/EV-p24 Ab2 signal tag solution). Therefore, 37 °C and 30 min were selected as the optimum incubation temperature and time in this study.

### 2.4. Characterization of Signal Amplification by AuNPs/EV-p24 Ab2 Using Different Labels

**Figure 7:**
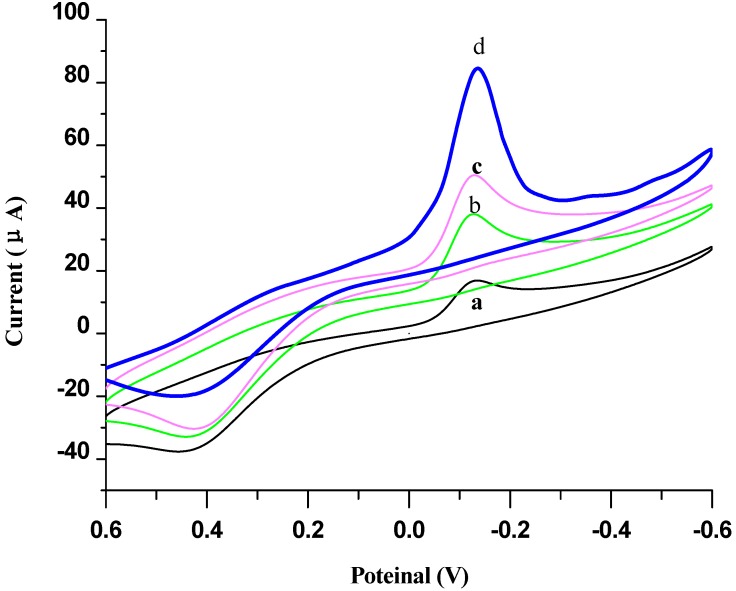
Cyclic voltammograms of the electrochemical sandwich immunosensor toward 5 ng/mL p24 in the absence (**a**) and in the presence of 5 mmol/L H_2_O_2_ in electrolytic cell in pH 7.0 PBS containing 5 mmol/L HQ by using various signal tags: (**b**) HRP-p24 Ab2; (**c**) EV-p24 Ab2; and (**d**) AuNPs/EV-p24 Ab2.

To further investigate the signal amplification effect of different signal tags, three types of detection antibodies were employed for the measurement of 5 ng/mL p24 antigen, such as HRP-p24 Ab_2_, EV-p24 Ab_2_ and Au NPs/EV-p24 p24 Ab_2_ (Figure 7b,c). Figure 7a was the background CV figure in the base solution of 5 mmol/L HQ and H_2_O_2_, whose reductive peak current was employed as substrate current *I*_0_.

As shown in Figure 7, the catalytic current (*I*_cat_) was generated mainly due to the labeled HRP, which can catalyze the reaction between H_2_O_2_ and HQ. Moreover, the use of AuNPs/ EV-p24 p24 Ab_2_ as the signal tag exhibited the maximum current shift over the other labeled tags, which were HRP-p24 Ab_2_ (b) and EV-p24 Ab_2_ (c). Upon addition of H_2_O_2_ into the PBS, the sandwich immunocomplex of MNPs-p24 Ab1/p24/ HRP-p24 Ab_2_ was dropped on the SPCE electrode, and the results showed that its reduction peak current increased dramatically, but the oxidation peak current decreased. The reduction peak current also increased when the concentration of H_2_O_2_ was increased, thereby displaying the obvious electrocatalytic behavior of HRP to the reduction of H_2_O_2_ [26]. These results indicated that the immobilized HRP on p24 Ab2 can catalyze the reduction of H_2_O_2_. All current values were shifted to more positive values in the full potential range when going from CV a to d, suggesting that from a to d, more and more HRP has taken part in the reaction between H_2_O_2_ and HQ. The reason might be that AuNPs could provide more specific surface area for increasing numbers of EV, which has a higher HRP enzyme marker density. When the AuNPs/EV-p24 Ab2 signal tag was incubated and reacted with the corresponding antigen, MNPs-p24 Ab1 on the surface of immunosensor, several hundred HRP molecules enter into the electrochemical immunoreaction and facilitate the catalytic oxidation of HQ by H_2_O_2_. As the data shows in Figure 7, the use of AuNPs/EV-p24 Ab2 tag (ΔI = 88.82 µA, Figure 7c) resulted in a much higher current response (approximately four-fold greater) than that of routine ELISA detection antibody HRP-p24 Ab2 (Δ*I* =15.78 µA, Figure 7a). It indicated that the immunosensor based on the AuNPs/EV-p24 Ab2 signal tag can generate substantial signal amplification and greatly enhance the sensitivity of the immunoassay.

### 2.5. Calibration Curve of the Immunosensor

**Figure 8:**
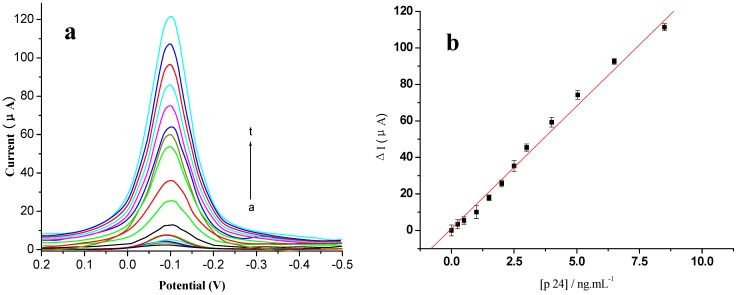
Voltammogram of the modified electrode using different concentrations of antigen (0~10 ng/mL, respectively) (**a**) DPV curves; (**b**) plot of ΔI *vs*. the concentrations of p24. All experiments were carried out in 0.01 mol/L PBS, 0.1 mol/L KCl (pH 7.4) and added 5 mmol/L HQ and H_2_O_2_, at a potential range of 0.2 V to −0.5 V, *vs.* SCE. Scan rate 50 mV/s.

Under the optimal conditions, the immunosensor was used for the detection of various concentrations of the p24 antigen. Differential pulse voltammetry (DPV) was used for quantification, because its assay has a higher sensitivity than CV. As shown in Figure 8a, the catalytic peak by DPV in the presence of p24 (curve b) was higher than that in the absence of p24 (curve a), and increased gradually as increasing concentration of p24 (curve b→t). The standard calibration curve for p24 detection was shown in the inset of Figure 8b.

The change of peak intensity ((Δ*I*) increased with an increase in the p24 concentration ([p24]) and showed a linear range from 0.001 to 10.00 ng/mL (*n* = 5). The regression equation was Δ*I* = 13.67 × [p24] + 0.47 (ng/mL), with a correlation coefficient *r* of 0.9989. The detection limit was estimated to be 0.3 pg/mL. According to the linear equation, we could detect p24 concentration quantitatively. Higher p24 levels could be detected by an appropriate dilution with PBS. Table 1 provides a comparison of the analytical properties of various p24 immunosensors and immunoassays. The results showed that the immunosensor has a higher sensitivity than the other immunosensors reported.

**Table 1:** Comparison of analytical properties of various p24 immunosensors or immunoassays.

Assay Method	Linear Range (ng mL^−1^)	LOD (ng mL^−1^)	Signal Antibody	Reference
ELISA	3–60	1	HRP-labeled p24 Ab2	Followed the kit’s instructions
Electrochemical immunoassay	0.01~60.00	0.0064	Gold nanoparticles (GNP)/multi-walled carbon nanotubes (CNTs)/acetone-extracted propolis (AEP) nanocomposite	[2]
	0.01–35	0.003	DNA modified gold nanoparticles	[1]
This method	0.001–10	0.0005	Au/EV-p24 Ab2	This work

### 2.6. Specificity, Reproducibility and Stability of the p24 Immunosensor

To investigate the specificity of the fabricated immunosensor, the immunoassay was performed in a 0.2 ng/mL HIV p24 sample solution containing the interfering substances of 2.5 ng/mL HIV gp 36 protein, 5 ng/mL HIV gp 160 protein, 10 ng/mL HIV antibody and 500 ng/mL human IgG. All the concentrations are normal concentration in an HIV-infected person. The mixed sample solution was measured by the immunosensor and the results were compared with those of the standard p24 solution (0.2 ng/mL). The catalytic current variation due to the interfering substances was less than 5.0% without interferences, indicating the selectivity of the immunosensor was satisfactory.

The reproducibility of the immunosensor was evaluated by detecting 0.2 ng/mL p24 with five immunosensors prepared equally. The relative standard deviation of the measurements for the five immunosensors was 2.5%, indicating the excellent precision and reproducibility of the immunosensor.

Regeneration of the immunosensor was examined by detecting 0.2 ng/mL p24 with the same immunosensor. The immunosensor was regenerated by dipping into 0.5 mol/L HAc-methnol buffer solution (pH 4.5) for 10 min to break the antibody–antigen linkage. The consecutive measurements were repeated eight times, then an average recovery of 95.4% and an intra-assay RSD of 4.5% was acquired. The results demonstrated that the proposed immunosensor could be regenerated and used for at least 20 times.

After the immunosensor was stored at 4 °C over two weeks, it was used to detect the same p24 concentration. The response of the immunosensor retained about 90% of its initial value, demonstrating that the immunosensor had a good stability. Thus, the developed immunosensor is an appropriate tool for the detection of p24 based on the obtained results.

### 2.7. Application of the Immunosensor in HIV Serum Samples 

In order to investigate the applicability and reliability of the prepared immunosensor for clinical applications, recovery experiments were performed by standard addition methods in six human serum samples from Nanfang Medical Hospital in China. Results were listed in Table 2, and showed an acceptable recovery from 90% to 115%. The results listed in Table 2 were also compared with the traditional ELISA method. The relative deviation of the measured data was between 1.3% and 3.1%, indicating that this method was precise in its p24 detection, and the results are in good agreement with the ELISA method, thus suggesting that the new method is reliable and exact. The results also revealed that the developed ECL immunosensor may provide an efficient tool for ultrasensitive determination of p24 in human serum samples.

**Table 2:** Experimental results comparison of two methods obtained in serum samples (*n* = 3).

Serum Samples	1	2	3	4	5	6
ELISA (ng/mL)	5	22	40	ND	ND	ND
Immunosensor (ng/mL)	5.2	24	42	0.1	0.5	2
Relative deviation (%)	1.3	2.5	3.1	2.3	1.3	2.1

Note: ND: not detected.

## 3. Experimental Section

### 3.1. Apparatus and Regents

HIV p24’s ELISA kit was supplied by Coulter Immunology (Hialeah, FL, USA). EnVision™ Detection Kit was supplied by Gene Tech Company Limited (Shanghai, China). Bovine serum albumin (BSA, 96%–99%), tetraethyl orthosilicate (TEOS), cetyltrimethyl ammonium bromide, and ammonia were purchased from Chinese Chemical Reagent Co. Ltd. (Shanghai, China). 1-ethyl-3-(3-dimethyl-aminopropyl) carbodiimide (EDC), amine-reactive N-hydroxysuccinimide (NHS) esters, gold chloride (HAuCl_4_) were from Aladdin chemistry Co. Ltd. (Shanghai, China). The hydroxyl of phenol (HQ) and H_2_O_2_ were obtained from Sinopharm Group Chemical Reagent Company Ltd (Shanghai, China). Phosphate buffer saline (PBS, 0.01 mol/L pH 7.4) containing 0.1 mol/L KCl was used to prepare the protein solution. The blocking buffer solution was PBS (pH 7.4) containing 3% (w/v) BSA. Tween-PBS buffer (0.01 mol/L pH 7.4) containing 0.05% (w/v) Tween-20 was used as the washing solution. All other reagents were of analytical grade and were used without further purification. Double-distilled water was used throughout the study. All experiments were carried out at room temperature.

Cyclic voltammetry (CV) and differential pulse voltammetry (DPV) were performed by an electrochemical analyzer CHI 660 electrochemical analyzer (Shanghai Chen hua Instrumental Corp, China). A conventional three-compartment electrochemical cell consisting of a platinum wire auxiliary electrode, a saturated calomel reference electrode and a modified GE (2 mm diameter) as a working electrode was used. The sizes of the nanoparticles were estimated by TEM (H-7650, Hitachi Instruments, Tokyo, Japan). The surface topographic features and composition of different modified electrodes were characterized using SEM (S3400N Hitachi), UV-VIS and XRF.

### 3.2. Preparation of EnVision-p24 Antibody Copolymer (EV-p24 Ab2)

Equal volumes of p24 Ab2 and EnVision antibody complex were mixed and then stored overnight at 4 °C. After centrifuging, the EV-p24 Ab2 was stored at 4 °C for further use.

### 3.3. Preparation of MNPs–p24 Ab1 Capture Probe and AuNPs/EV-p24 Ab2 Signal Tags

Fe_3_O_4_@SiO_2_ (MNPs) nanostructures were prepared according to a method [27] analog to the classical Stöber process [28]. Typically, Fe_3_O_4_ magnetic nanoparticles (0.5 g) were added into 0.1 mol L^−1^ hydrochloric acid (100 mL) and was shocked under the ultrasound for 10 min. The separated magnetic particles (by external magnet) were added into anhydrous ethanol (100 mL) with a certain amount of CTAB surfactant and ammonia. The solution was uniformly mixed and stirred under ultrasonic dispersion for 30 min, and then in a 35 °C water bath; a certain amount of TEOS was added drop-wise to the above solution and reacted for 20 min. After the reaction was complete, the surfactants and surplus ammonia were separated in order to remove the non-magnetic substances. The product was then placed in acetone to reflux for 48 h at 60 °C. The resulting precipitates were separated by external magnet and washed three times with double-deionized water and ethanol, respectively, in order to obtain the Fe_3_O_4_@SiO_2_ NPs (MNPs).

The capture probe based on p24 Ab1 coated on Fe_3_O_4_@SiO_2_ NPs was prepared as follows: the MNPs’ surface was deactivated by ethanolamine (1 mol L^−1^ at pH 8.5) and then activated by EDC (0.2 mol L^−1^)/NHS (0.05 mol L^−1^). After the linked reaction by EDC/NHS, the p24 Ab1 antibody was immobilized on the magnetic MNPs microspheres and thus, the p24 Ab1-modified MNPs were formed as capture probes (MNPs-p24 Ab1), which could possess strong combining capacity with p24 antigen and obviously promote the enrichment and separation ability even for the smaller p24 levels in serum when passing through its surface.

The signal tags (AuNPs/EV-p24 Ab2) were prepared by the following steps: Firstly, peptide–AuNPs conjugates were prepared by mixing protein A (1 mg mL^−1^) and AuNPs (18 nm diameter, 1 mg mL^−1^) in pH = 7.0 PBS solutions for 2 h, and then centrifuged at 10,000 rpm. Thus, AuNPs modified by protein A (AuNPs-protein A) were acquired in the precipitation. Secondly, EV-p24 Ab2 antibodies were immobilized on the AuNPs–protein conjugates. EV-p24 Ab2 solution (1 mg mL^−1^) was initially adjusted to pH 8.2 using sodium carbonate, and then, the original AuNPs–protein A solution (1.0 mL) was added into the mixture and incubated for 12 h at 4 °C with slight stirring. After separation at 10,000 rpm, the obtained AuNPs-Ab2 were incubated with 1.0 wt % HRP (1 mL) for 1 h to block the nonspecific sites on the uncovered surface of AuNPs. The synthesized AuNPs/EV-p24 Ab2 signal tags were stored in pH 7.4 PBS at 4 °C when not in use.

### 3.4. Fabrication of the Immunosensor

**Scheme 1:**
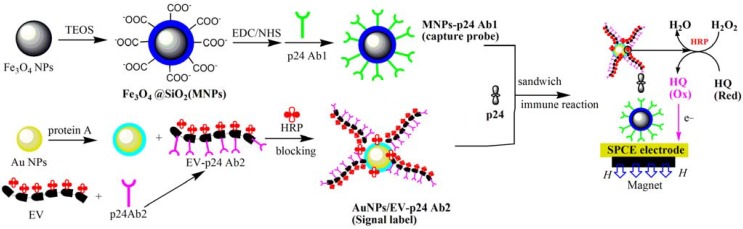
Illustration of the fabrication and detection procedure of the immunosensor.

The MNPs-p24 Ab1 was immobilized onto the SPCEs by a magnet in advance. The reaction cell contained 100 μL of MNPs-p24 Ab1 conjugations (1:10 dilution with PBS containing 1 wt % BSA). The electrode was then incubated with different concentrations of p24 for 15 min at 37 °C. After incubation, the wells were washed six times with pH 7.0 PBS containing 0.05% W/V Tween-20 and then incubated with 100 μL of 1 mg mL^−1^ Au /EV-p24 Ab2 signal tags at 37 °C for 15 min. After the wells were rinsed, 100 μL of 5 mmol L^−1^ HQ and H_2_O_2_ mixture solution was added into the well, and the catalytic current of the electrode was recorded. The procedures are shown in Scheme 1.

## 4. Conclusions

Herein, we present an ultrasensitive and disposable amperometric immunosensor that can detect pg/mL concentrations of HIV p24 protein. Its detection sensitivity was 1000 times higher than that of the routine ELISA method. The novel signal amplification strategy for the construction of an electrochemical sandwich immunosensor was based on a novel signal tag (AuNPs/EV-p24 Ab2) with a large amount of HRP labeling on it. Dilution studies using p24-spiked human plasma samples also indicated that the immunosensor is robust against the interference effects of a complex biological matrix. Moreover, the capacitive immunosensor assay is rapid (<30 min), label-free, and generates data in real-time, with a portable format in development. Therefore, the proposed immunosensor could provide a new approach for large-scale sample screening detection and early diagnosis of AIDS.
